# Boosting Immunity in Recipients of Live-Attenuated Zoster Vaccine

**DOI:** 10.1093/infdis/jiv481

**Published:** 2015-10-09

**Authors:** D. Scott Schmid

**Affiliations:** Herpesvirus Group and National VZV Laboratory, Centers for Disease Control and Prevention, Atlanta, Georgia

Roughly a million cases of herpes zoster (HZ) occur annually in US adults, and nearly one third of these patients will experience postherpetic neuralgia (PHN) lasting for ≥1 month [1]. The burden of HZ is strongly influenced by age: the incidence of HZ increases many fold throughout adulthood, and among adults who experience HZ, the risk that PHN will develop increases several fold more after the age of 60 years [1]. These conditions are often sufficiently severe to compromise daily lifestyle, and PHN pain is frequently refractory to treatment [1].

The initial report on the clinical trial for zoster vaccine indicated that it was partially protective in adults ≥60 years of age [2]. The placebo-controlled trial demonstrated that the vaccine prevented half of all HZ cases and 67% of PHN cases and reduced the burden of illness by 61% [2]. Two follow-up studies of the trial cohort have suggested a marked decline in HZ vaccine efficacy by 7–11 years after immunization [3, 4]. In one of the studies, vaccine efficacy had declined from 51% to 40% against HZ and from 67% to 60% against PHN 7 years after vaccination, although these differences were not statistically significant [3]. In the other study, protection had declined to 21% against HZ and to 35% against PHN 11 years after vaccination [4]. However, this longer-term study was conducted without a concurrent control group, so these results need to be verified. While neither of these studies evaluated concomitant declines in varicella zoster virus (VZV)–specific humoral and cell-mediated immune responses over time, it is well established that responsiveness to vaccines wanes with advancing age [5]. Although both arms of the acquired immune response likely contribute to anti-HZ immunity, there is solid evidence that cell-mediated immunity plays a particularly important role in resistance to HZ [6, 7].

The article by Levin et al in this issue of *The Journal of Infectious Diseases* [8] provides an important preliminary insight into the acquired immune responses to a second dose of zoster vaccine in persons ≥70 years of age. No serious vaccine-related adverse events were observed, although the authors did not mention this in their discussion. The response to a first dose of zoster vaccine was also evaluated in 3 age groups: 50–59 years, 60–69 years, and ≥70 years. VZV-specific immunoglobulin G (IgG) levels peaked at 6 weeks for all test groups. The IgG responses in all 4 groups were comparable, even for those receiving their first dose of vaccine at ≥70 years of age. Cell-mediated immune (CMI) responses were evaluated using enzyme-linked immunospot assays to measure interferon-γ (IFN-γ) and interleukin 2 (IL-2) secretion by effector and memory T cells. In contrast to the IgG response, significant differences were observed in the CMI responses of the 4 different study groups. As expected, the CMI responses in the group of individuals aged ≥70 years receiving their first dose were the least robust; however, persons aged ≥70 years receiving a booster dose 10 years after the first dose responded comparably to persons in the group aged 60–69 years. The booster in these individuals thus demonstrated a residual effect of the first dose. The strongest CMI responses were obtained from persons 50–59 years old receiving their first dose of vaccine. The authors speculate that booster doses might be expected to be more effective in adults receiving their first dose between the ages of 50 and 59 years.

Two studies of participants in the Shingles Prevention Trial indicated that CMI but not IgG levels correlated with reduced HZ morbidity [9–11]. Although those results imply a correlation of elevated VZV-specific CMI with protection, it should be stressed that the study reported here did not provide any clinical correlations of protective immunity. In addition, the effect of boosting was limited to a single interval of 10 years after immunization. While zoster vaccine is licensed for adults aged ≥50 years, the Advisory Committee for Immunization Practices (ACIP) recommends the vaccine for persons aged ≥60 years. A key consideration of the ACIP was suggestive evidence that protection wanes over time, raising the concern that vaccination at age 50 years would leave seniors unprotected decades later, when the burden of HZ and PHN are substantially greater. The important safety and immunogenicity results in the report by Levin et al raise the prospect that revaccination could help resolve this concern. As the authors point out, the clinical implications of these findings are not fully understood, but they support additional work to verify the benefits of revaccinating seniors against HZ at an appropriate interval after initial vaccination. Recent results indicating excellent protection against HZ following administration of an investigational glycoprotein-based vaccine bear watching, as well [12].
